# Concurrent Polycythemia of Undetermined Etiology and Smouldering Plasma Cell Myeloma

**DOI:** 10.1155/2018/8781721

**Published:** 2018-09-16

**Authors:** Roula Katerji, Chad A. Hudson

**Affiliations:** Department of Pathology and Laboratory Medicine, University of Rochester, Rochester, NY, USA

## Abstract

The combination of polycythemia and plasma cell myeloma occurring concurrently is very rare and few cases have been reported in the literature. Further, the vast majority of these cases are cases of polycythemia vera and myeloma. Here, we present a case of polycythemia of undetermined etiology and myeloma. The patient is a 48-year-old Caucasian male who was originally diagnosed with polycythemia of undetermined etiology. Twelve years later, when a bone marrow biopsy was performed in an attempt to determine the etiology of the polycythemia, findings diagnostic of plasma cell myeloma were discovered. Subsequent serum studies were also consistent with a plasma cell neoplasm, while evaluation for end-organ damage was negative. A battery of genetic and biochemical tests ruled out various congenital polycythemias, leading to a final diagnosis of polycythemia of undetermined etiology and smouldering plasma cell myeloma. This case highlights that while being unusual, polycythemia and plasma cell myeloma can occur concurrently, and, in this report, we discuss both entities and potential mechanisms of the pathophysiology of the concurrent presentation.

## 1. Introduction

Plasma cell myeloma (PCM) accounts for approximately 1.6% of all cancer cases and about 10% of all hematologic malignancies in the United States [1] and is characterized by the proliferation of malignant plasma cells and subsequent increase of a monoclonal paraprotein in serum. Studies usually done when suspecting myeloma include serum and urine electrophoresis, serum-free light chain assay, bone marrow aspiration and biopsy with karyotype and FISH analysis, measurement of serum levels of beta-2 microglobulin, albumin, and LDH, and imaging, including skeletal survey, MRI, CT, and/or PET. One of the two following criteria is required for a diagnosis of PCM:

1. Clonal bone marrow plasma cells ≥ 10% or biopsy-proven bony or extramedullary plasmacytoma and any one or more of the following signs of end-organ damage that can be attributed to the plasma cell neoplasm:
  (i) hypercalcemia, renal insufficiency, anemia, and bone lesions (CRAB criteria).
2. Clonal bone marrow plasma cells ≥ 10% or biopsy-proven bony or extramedullary plasmacytoma and clonal bone marrow plasma cells ≥ 60% or involved: uninvolved serum-free light chain ratio ≥ 100 or more than one focal lesion on MRI [2].

Patients that have ≥ 10% clonal bone marrow plasma cells but do not show signs of end-organ damage are classified as having a variant of PCM, smouldering (asymptomatic) PCM [2]. With anemia being part of the CRAB criteria for the diagnosis of PCM, it is not surprising that anemia is a common association and complication of PCM, occurring in more than two-thirds of PCM patients [3]. There are multiple proposed mechanisms for the concurrent anemia, including anemia of chronic disease, erythropoietin (EPO) deficiency due to renal problems, and chemotherapy effect upon initiation of treatment [3].

Conversely, polycythemia is an uncommon finding in PCM patients [4]. Polycythemia can be divided into two types, primary and secondary, based on the pathophysiology causing the increased hematocrit. Diseases in which the increased hematocrit is due to a mutation that leads to an intrinsic alteration in red blood cells are known as primary polycythemias. Polycythemia vera (PV) is the archetypal primary polycythemia and is almost always caused by a somatic mutation (V617F) in the JAK2 gene that leads to constitutive JAK2 activation and downstream signaling. Primary familial polycythemia (PFP) is caused by an autosomal dominant mutation in the EPO receptor gene [5]. In PFP, the findings are typically isolated erythrocytosis and normal hemoglobin oxygen affinity, with EPO being low to low normal.

Alternatively, secondary polycythemias are caused by increased production of EPO, whether it be the result of chronic hypoxia, due to either diseases such as COPD and chronic sleep apnea or environmental conditions such as living at a high altitude, an EPO-producing neoplastic disease, such as renal cell carcinoma, von Hippel Lindau (VHL) disease, pheochromocytoma, and adrenal adenoma, or a disorder of hypoxia sensing, which are predominantly caused by mutations in VHL or hypoxia-inducible factor (HIF). Chuvash polycythemia, first described in the Chuvash region in Russia, is a rare autosomal recessive congenital polycythemia caused by a VHL^R200W^ germline mutation in the VHL gene. The VHL protein marks the alpha subunits of HIF-1 and HIF-2 for destruction by the proteasome when oxygen levels are normal. In Chuvash polycythemia, the mutation causes impaired recognition of the alpha subunits of HIFs by VHL, leading to impaired degradation of HIF in normoxic conditions, which in turn leads to an aberrant upregulation of the body's hypoxic response and increased EPO production [6].

## 2. Case Presentation

A 48-year-old healthy male presented with a hemoglobin level of 21 mg/dl and an elevated hematocrit (63%). The patient had an increased hematocrit (64%) 12 years ago, leading to clinical suspicion of polycythemia, although the patient was quickly lost to follow-up. At that time, JAK2 mutational testing was negative. At the current presentation, the patient reported fatigue, headache, blurred vision, and excessive sweating. He declined both a history of living at high altitude and smoking. Exogenous EPO use was also excluded. Past medical history includes atrioventricular block requiring pacemaker insertion, hypertension controlled with lisinopril, mild depression managed with citalopram, and erectile dysfunction treated with sildenafil. On physical examination, the patient's vital signs were as follows: blood pressure: 140/100 mmHg; heart rate: 97; respiratory rate: 19 (with excessive redness in the face); BMI: 25.85 kg/m^2^; SpO2: 100%.

### 2.1. Labs and Treatment

  WBC: 3.6 x 10^9^/L
  HGB: 19.4 g/dL
  Hct: 63 %
  MCV: 85 fl/cell
  MCH: 26 pg
  MCHC: 31 g/dL
  RDW: 25.7 %
  PLT: 132 x 10^9^/L
  Creatinine: 1.2 mg/dl (normal range: 0.6-1.2)
  Carboxyhemoglobin: 1.5% (normal range: 0-1.4%)
  EPO: 687 mU/ml (normal range: < 29.5)

CT scans of the chest and abdomen showed no evidence of malignancy, while MRI of the head was negative. Subsequent genetic/biochemical testing ruled out congenital polycythemias.

**Figure 1:**
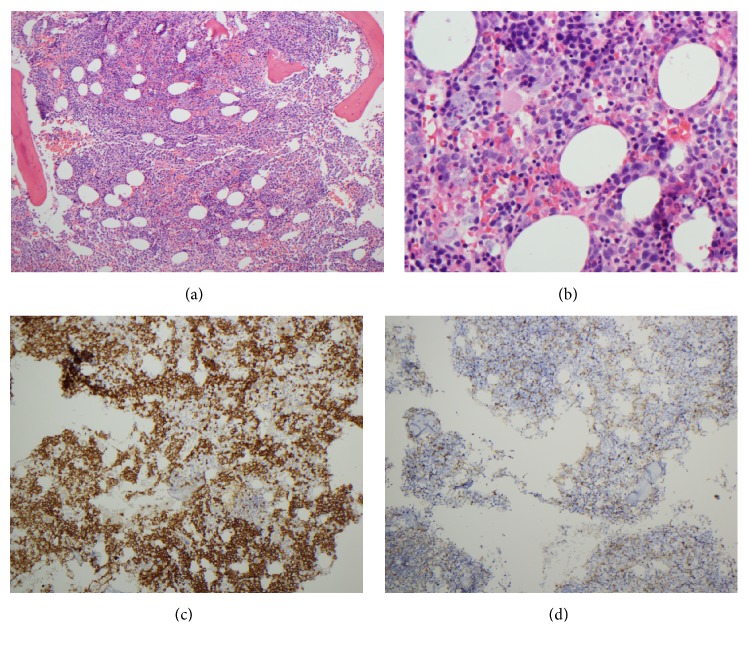
Bone marrow biopsy. (a, b) Representative areas of hematoxylin and eosin-stained sections of the bone marrow biopsy ((a) 10x, (b) 40x). (c) Immunohistochemical (IHC) staining for the erythroid marker CD71 (10x). (d) IHC stain for the myeloid marker MPO (20x).

**Figure 2:**
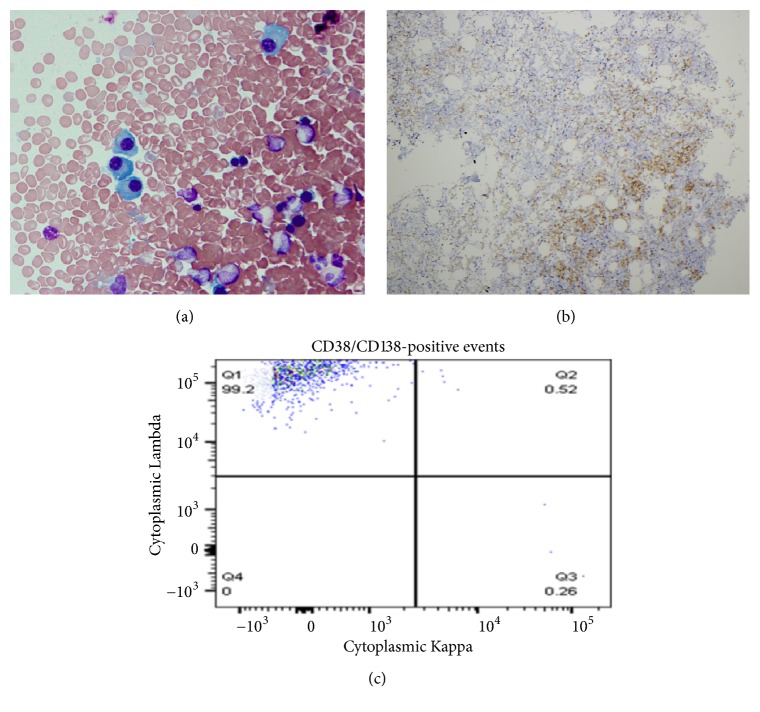
Evidence of a plasma cell neoplasm. (a) Touch prep of the bone marrow biopsy showing numerous plasma cells (arrow, 50x). (b) IHC staining for the plasma cell marker CD138 (10x). (c) Flow cytometric dot plot of CD38/CD138-positive plasma cells from the bone marrow aspirate showing a monotypic lambda-restricted plasma cell population.

Due to the polycythemia having no obvious etiology, a bone marrow aspiration and biopsy were performed (Figures 1(a) and 1(b)). The core biopsy showing a markedly decreased myeloid: erythroid ratio with CD71-positive erythroid precursors comprising ~80% of marrow cellularity (Figure 1(c)) and MPO-positive myeloid cells comprising < 10% of cells (Figure 1(d)). Surprisingly, the touch prep revealed increased plasma cells (16%, Figure 2(a)), and CD138 immunohistochemical staining highlighted the increased plasma cells arranged in clusters in the core biopsy (~15% of cellularity, Figure 2(b)). Flow cytometry demonstrated the bone marrow aspirate contained a monotypic lambda-restricted CD38/CD138-positive plasma cell population (Figure 2(c)). Karyotype analysis of the aspirate revealed a normal male 46,XY karyotype, while FISH revealed an IGH/CCND1 rearrangement (t(11;14)), solidifying a diagnosis of plasma cell myeloma. Subsequent serum studies showed increased serum lambda light chains (41.58 mg/dl; normal range: 0.57-2.63 mg/dl) and a kappa: lambda ratio of 0.02. Immunofixation revealed an IgD lambda paraprotein that was too small to quantitate.

### 2.2. Treatment

The patient was phlebotomized until Hct dropped below 50, which led to the resolution of symptoms. The patient has not been treated for the smouldering PCM.

## 3. Discussion

The combination of polycythemia and PCM is very rare, with most of the cases reported in literature being cases of concurrent PV and PCM [4]. The exclusion of these PV cases leaves a total of six cases in the literature (including this current case of the smouldering variant of PCM which was deemed to be not to be PV due to the absence of the archetypal V617F mutation and elevated EPO levels even though exon 12 mutational studies were not performed), all of which can be characterized as PCM with concurrent polycythemia of undetermined etiology. In three of these cases, the polycythemia significantly preceded the diagnosis of PCM (as in our case), while, in the other two cases, the diseases were diagnosed concurrently [7]. The initial treatment in all of the cases was phlebotomy, with no known treatment for the myeloma in all of the cases in which the polycythemia preceded the myeloma. Interestingly, in the two cases in which the diseases presented concurrently, treatment of the myeloma led to amelioration of both the myeloma and the polycythemia, suggesting an interconnected mechanism [7, 8].

While the combination of polycythemia and PCM is rare, there are rare syndromes that are characterized by both polycythemia and a monoclonal gammopathy, and these syndromes should always be in the differential in patients with both a monoclonal plasma cell process and polycythemia. Polyneuropathy, organomegaly, endocrinopathy, monoclonal protein, and skin changes (POEMS) syndrome is a rare syndrome with the following diagnostic criteria: major criteria: polyneuropathy, monoclonal plasma disorder, Castleman's disease, sclerotic bone lesions, increased vascular endothelial growth factor; minor criteria: organomegaly, extravascular volume overload, skin changes, endocrinopathy, papilledema, thrombocytosis, and polycythemia [2, 9]. A diagnosis requires the fulfillment of at least three major criteria, two of which must be polyradiculoneuropathy and a clonal plasma cell disorder, along with at least one minor criterion [9]. It has been suggested that the manifestations of POEMS are the result of a marked activation of the proinflammatory cytokines IL-1beta, IL-6, and TNF-a along with an associated decrease in the presumptive anti-inflammatory cytokine TGF-beta [10].

Another rare syndrome with erythrocytosis and clonal plasma cells is telangiectasias, elevated EPO and erythrocytosis, monoclonal gammopathy, perinephric fluid collections, and intrapulmonary shunting (TEMPI) syndrome [2, 11]. The pathogenesis of TEMPI syndrome is still not clear, but it has been hypothesized that the monoclonal protein leads to a paraneoplastic syndrome and increased erythropoietin production, similar to the mechanism seen in solid tumors [11].

In our case, these two syndromes were briefly considered, but as the patient lacked any symptomology other than those due to the polycythemia, criteria were not fulfilled for either of the syndromes. Moreover, the clonal plasma cell process in POEMS usually does not reach the level of PCM (it usually is characterized as a monoclonal gammopathy of undetermined significance (MGUS)) [12], and EPO levels are usually decreased in POEMS [13]. Likewise, the clonal plasma cell process in TEMPI is usually at the level of MGUS [14].

One theory that has been posited in attempt to explain this rare association between PCM and polycythemia is that the deposition of monoclonal light chains causes damage in kidney tubules leading to hypoxia, resulting in increased EPO production. The cases mentioned above in which treatment of the PCM ameliorated the polycythemia are compatible with this theory [7]. This theory seemingly would require the plasma cell malignancy to precede the polycythemia. While at first glance this does not seem congruent with the case we present here since the polycythemia was diagnosed first, it is impossible to know when the patient's asymptomatic monoclonal plasma cell disorder began.

In conclusion, this case highlights that while myeloma is usually associated with anemia, the presence of polycythemia does not rule out plasma cell myeloma, whether it be symptomatic or smouldering. In fact, in rare cases, myeloma can occur concurrently with polycythemia of undetermined etiology, and the possible interconnection between the two entities is an intriguing area for further studies.

## Data Availability

The data used to support the findings of this study are included within the article.
